# Truth and Error in Vaccination

**Published:** 1892-11

**Authors:** 


### HOMŒOPATHIC PHYSICIAN,

A MONTHLY JOURNAL OF

HOMŒOPATHIC MATERIA MEDICA AND CLINICAL MEDICINE.

"If our school ever gives up the strict inductive method of Hahnemann, we are lost, and deserve only to be mentioned as a caricature in the history of medicine."—CONSTANTINE HERING.

Vol. XII. NOVEMBER, 1892. No. 11.

### EDITORIAL.

TRUTH AND ERROR IN VACCINATION is the title of the first article in this month's issue of THE HOMŒOPATHIC PHYSICIAN. This article by Dr. Stuart Close was read before the International Hahnemannian Association, and, as can be seen, is a most reasonable and interesting review of the subject.

The point made by the learned writer is given in his article in italics, and may be here repeated: *Vaccination owes any degree of efficiency it may possess, either as a prophylactic, or as a therapeutic measure, to its homœopathicity to the case in which it is employed, and the extent of the protection afforded, or of modification secured, is proportioned to its degree of similarity.*

Here is the key to the whole controversy. It is this solution of the question which, though so obvious, is neglected by many in the homœopathic school, and totally unknown to the practitioners in the other school.

Any substance in the world that is capable of making any impression upon the human system is likely to be the simillimum for some sick condition of some human being. It then becomes a lottery as to whether that particular simillimum shall reach that particular diseased individual to whose case it is adapted. Sometimes by chance the meeting does take place and the individual is cured. Oftener it does not take place because mankind in general have no knowledge whatever of the law of the similars, and therefore have no provings to guide them.

A quack medicine made up in vast quantities and sold to people all over the world, must somewhere, at some time, meet the case for which it is the similar and produce a cure. This cure, heralded by the vender of the medicine, causes an increased demand for his nostrum and so other cases here and there are benefited while the vast majority have no relief because it was not the simillimum to their cases.

Vaccine virus introduced into the circulation of people promiscuously must prove the simillimum here and there, and consequently bring about protection in these cases. Thereupon the practice of vaccinating everybody in the world is attempted upon the evidence afforded by the few successful cases.

The same remarks apply to Koch's tuberculous lymph.

Consequently, we need not be surprised when we hear of cures being performed by various kinds of proprietary medicines, by herbs, "old women's remedies," inoculations with all manner of lymphs, nosodes, and other agents. Neither should we be puzzled by the success of apparently opposite and contradictory methods of treatment in pathological cases that are similar or apparently identical.

The failures with all modes of treatment in certain cases where previous experience would teach us to expect success, are not to be wondered at, either. He who understands the law of the similars and the proving of drugs has the key to unlock the chamber of difficulty, and to reconcile the contradictions.

A general acceptance of the truth of the law of similars would sweep away much dispute, and afford a guide through the labyrinth of phenomena which, as now accumulated, causes only confusion.

Drugs which to-day are loudly praised and universally used, and to-morrow are cast aside and forgotten, would then take a permanent place in the materia medica, their sphere of usefulness sharply defined.

The homœopathic principle, when properly developed, will enable the practitioner to predict the sphere of usefulness of any drug. Indeed, Hahnemann actually did make such predictions successfully, and when it becomes a universal experience, medicine will enter the domain of the exact sciences, a position which its votaries at present despair of its ever attaining.

